# The Fifth Annual Report of the Registrar-General of Births, Deaths and Marriages in England

**Published:** 1844-07-01

**Authors:** 


### The Fifth Annual Report of the Registrar-General of Births, Deaths, and Marriages in England. 8vo. pp. 324. London, 1843.

Since the first appearance of these Reports we felt it our bounden duty earnestly to recommend their attentive perusal to the members of the medical profession. The vast fund of valuable and practical precepts contained in them, whilst they evince the great skill and high scientific attainments of the gentlemen engaged in this department, cannot fail to prove of incalculable benefit not merely to those to whom the prevention and treatment of disease are more immediately committed, but to the public generally. Every page of the Report satisfies us that a steady advance is making towards the attainment of a more perfect system of registration, and that the attention of the Registrar-General is intently directed to the best means of removing all difficulties. From the very first appearance of these Reports, an obvious improvement was perceptible in every succeeding volume. In none of these Reports, however, is this progressive improvement more decided or marked than in the present. It contains the Report of the Registrar-General, and the returns on which this report is founded, the marriages births and deaths in four years—the present state of mortality, both over the whole country, and in different districts and different towns—the mortality at different ages—the mode of construction of Life-Tables—the principles on which they are constructed—their uses and mode of application for various purposes.

To this is added an Appendix, containing Mr. Farr's Letter to the Registrar-General, with accompanying Papers and Tables. The first Paper commences with the Construction of Life-Tables, in which he employs the differential method. Then we have a Paper headed *Public Health*, containing details of the zymotic and sporadic diseases which prevailed during the year 1841, and an elaborate account of the deaths by different causes in the four years 1838-41. Then an Abstract of the Causes of Death registered in the year 1841 in the towns and the open country, with an elaborate detail of the causes of the high mortality in town districts. Next follows an account of the causes of the mortality at different periods of life, &c. &c. Having now presented a summary of the contents of this excellent volume, we proceed to our analysis of those contents.

The present volume commences with a General Abstract of the Births, Deaths, and Marriages registered in England, during the year 1841. In order to show the progress of registration, and the changing state of the population, the Births, Deaths, and Marriages of the preceding years are subjoined:

| | 1838 | 1839 | 1840 | 1841 |
|---|---|---|---|---|
| Marriages . . | ... | 123,166 | 122,665 | 122,496 |
| Births . . . | ... | 492,574 | 502,303 | 512,158 |
| Deaths . . . | 342,547 | 338,979 | 359,634 | 343,847 |

The Registrar-General seems determined to make the future series of his annual abstracts to terminate on December 31st, instead of June 30th as was heretofore done. The Marriages in 1841 were 1 in 130, the Births 1 in 31, the Deaths 1 in 46 of the population; the average of the two preceding years having been of Marriages 1 in 127, Births 1 in 31, Deaths 1 in 45. The Marriages diminished slightly in number every year; from 123,166 in 1839 to 122,496 in 1841; from 1 in 126 in 1839 to 1 in 128 in 1840, and 1 in 130 in 1841. Thus in the three years 1625, 1597, 1574 males in 100,000 were married, or less by 51 in 1841 than in 1839; whilst in the same three years 1553, 1526, and 1504 women in 100,000 were married; or less by 49 in the year 1841 than in 1831. In several cases the fluctuation in the number of marriages was observed to coincide with the depression or prosperity of industry or trade, and indicates with considerable accuracy the view which the people took of their own circumstances, and the greater or less immediate facility for providing for the support of families. The great good sense displayed by the working classes in England with respect to marrying cannot be too much recommended. Would that the same prudence and the same caution were observed among the corresponding classes in Ireland, among whom marriages are contracted with such precipitancy, and such little thought respecting the means of supporting their young families, that but for the well-known and truly proverbial devotedness and affection of the poor Irish peasant for his offspring, one might be disposed to think that in the affair of matrimony the principle which guided these wretched people was, "*solamen miseris socios habuisse malorum.*"

The greatest number of marriages (36,542) occur in Autumn, and the smallest number (25,174) in Winter: the difference between these extremes is 11,368, and the four seasons stand in the following order:—

| | *Winter.* | *Summer.* | *Spring.* | *Autumn.* |
|---|---|---|---|---|
| | January, | July, | April, | October, |
| | February, | August, | May, | November, |
| | March, | September, | June, | December, |
| Average (1839-41) | 25,174 | 29,502 | 31,559 | 36,542. |

"The marriages in Winter (25,174) are to those in Spring (31,559) very nearly as the marriages in Summer (29,502) are to the marriages in Autumn (36,542): and the marriages in Winter are to those in Summer, as the marriages in Spring to the marriages in Autumn." It will be at once evident that the latter of these proportions is included in the former, and directly inferrible from it by an *alternation* of ratios, and therefore need not have been stated. In fact its being stated involves a tautology. The marriages in the four quarters are thus in geometrical proportion nearly; whence the marriages in any three being given, the number of marriages in the other quarter can be deduced from them by the common rule of three within a few hundreds; or, as the terms are nearly in arithmetical proportion also, by adding two terms and subtracting the third. The regularity in the numbers registered in each of the three years indicates the operation of constant causes, or such as fluctuate only in the same way as those which adjust the proportion of marriages to the population. In order to distinguish the number of persons in the community who marry from the mere number of marriages, the Registrar-General had an abstract made of the number of widows and widowers re-married in the last half year of 1841. Of 65,498 women married, 5888, or 9 in 100, were widows; of the same number of men 8476, or 13 in 100, were widowers. The average annual number of marriages in the three years 1839, 1840, 1841, was 122,777; and it would follow that 106,975 men, and 111,765 women, or 218,740 persons marry every year.

The proportion of re-marriages is greatest in the metropolis, and in the North-Western, Western, and York Divisions, where the mortality is highest.

There were married under the age of twenty-one 5362 men, and 16,285 women; the proportion of minors (4·38 per cent. of the men, and 13·29 per cent. of the women) is below the averages of preceding years.

*Births.*—In 1841, 512,158 births were registered; in 1840, 502,303; and in 1839, 492,574; this increase, therefore, was 9855 in 1840, and 9729 in 1839; or 19,584 in two years. If the births had increased at the same rate as the population, the increase would have been about 14,000 in the two years: the excess of 5584 over this number may, I think, be fairly ascribed to the greater efficiency of this branch of registration. The births registered were to the deaths in 1841 as 149 to 100, or as 3 to 2. This proportion falls very far short of the ascertained annual increase of the population in the ten years 1831-1841. The greatest number of births is registered in the Winter quarter: the smallest in the Summer.

*Illegitimate Births.*—From the Registers of Births it would appear that 1 in 16 of the children born in England is not born in wedlock. The

Registrar-General sees no grounds for supposing that less than 64 in 1000 English children are illegitimate. The proportion in France is 71 in 1000. In the Fourth Annual Report it was shewed that the proportion of boys to girls born in England was 10,486 to 10,000—the proportion of boys has been ascertained by several to be greatest among legitimate children. In France, for instance, the boys are to the girls born as 106·4 to 100; but among illegitimate children the proportion is 104·4 to 100. The present return gives a result quite the reverse; of the legitimate births the boys are to the girls as 105·4 to 100; of illegitimate births the boys are as 108 to 100.

*Deaths.*—The deaths in the year ending June 30th, 1841, amounted to 355,655; in the year ending Dec. 31st, 1841, to 343,847; so that, although the two series of abstracts comprise the Winter and Spring quarters of 1841, the difference in the sums of the annual deaths is 11,808. On comparing the deaths in 1840 and 1841, there will be found a decrease of 15,787. The deaths were more numerous (99,069) in the Winter of 1841 than in the Winter of any preceding year, but in the Spring the decline commenced, which reduced the mortality in the following quarters below the mean mortality of the four years. Up to the year 1843 the deaths in the Summer quarter rose regularly from 72,791 to 80,822, in the Autumn from 80,833 to 89,630; the deaths in Winter except in 1839, when they were below the average number. The deaths in the four Winter quarters were 385,764, in the four Summer quarters 305,333; the deaths in the Springs 355,248, in the Autumns 338,662. With respect to the table, which is here given, and which shews the influence of the seasons very distinctly, before it is applied it requires correction for the different duration of the seasons, and for the increase of population. The four Winter quarters comprised 361 days, the Spring quarters 364 days, the Summers 368 days, and the Autumns 368 days; the population increased at the rate of 1·334 per cent. annually. If the quarters had contained the same number of days, and the population had been stationary, the deaths would have been nearly as follows:—

| | | | | |
|---|---|---|---|---|
| Four Winters | (365¼ days) | 391,059 | Quarterly averages (91 $\frac{81}{100}$ days) | 97,765 |
| Four Springs | ,, ,, | 356,565 | ,, ,, | 89,141 |
| Four Summers | ,, ,, | 302,827 | ,, ,, | 75,707 |
| Four Autumns | ,, ,, | 334,556 | ,, ,, | 83,639 |
| Mean . . . | | 346,252 | Mean . . . | 86,563 |

By observing the great excess of deaths in Winter in the above summary the fatal effects of cold are seen in a striking light. The average corrected number of deaths in the seasons would be—

| *Winter.* | *Spring.* | *Summer.* | *Autumn.* |
|---|---|---|---|
| 97,765 | 89,141 | 75,707 | 83,639 |

By transposing the Autumn and Summer terms, the law is discovered, which has regulated the mortality of the seasons, thus—

| | *Winter.* | | *Spring.* | | *Autumn.* | | *Summer.* |
|---|---|---|---|---|---|---|---|
| | 97,765 | : | 89,141 | : : | 83,639 | : | 75,707 |
| Differences | | 8,624 | | 5,502 | | 7,932 | |

The terms are in proportion; the product of the two extremes being nearly equal to the products of the means, or the deaths in Winter are to the deaths in Spring, as the deaths in Autumn to those in Summer. The proportion is perceived when no correction has been made in the quarterly deaths registered.

| | *Winter.* | | *Spring.* | | *Autumn.* | | *Summer.* |
|---|---|---|---|---|---|---|---|
| | 96,441 | : | 88,812 | : : | 84,666 | : | 76,333 |
| Differences . | 7,629 | | | 4,146 | | 8,333 | |

Admitting that this law should continue to prevail, as the proportion is also nearly arithmetical, a very close approximation to the average number of deaths in the whole year may be deduced either from the deaths registered in Spring and Autumn, or in Summer and Winter; thus the average annual deaths in the four years, 1838—41, was 346,252; the deaths in Spring and Autumn were 173,478; and twice that number gives 346,956, only 704 above the yearly average; while the deaths in Winter and Summer, multiplied by 2, give 345,548, or 704 below the annual average.

To exhibit the order in which marriages, births, and deaths take place more evidently, the Registrar-General annexes a summary view of the series of facts, which appear to be governed by the influence of the seasons according to the same law of proportion.

Relative number (corrected for inequality of time) of marriages, births, and deaths, in the seasons of the year—

| | | | | |
|---|---|---|---|---|
| | *Autumn.* | *Spring.* | *Summer.* | *Winter.* |
| Marriages, | 36,306 | 31,355 | 29,634 | 25,482 |
| | *Winter.* | *Spring.* | *Autumn.* | *Summer.* |
| Births . | 131,257 | 129,677 | 121,053 | 120,356 |
| | *Winter.* | *Spring.* | *Autumn.* | *Summer.* |
| Deaths . | 97,765 | 89,141 | 83,639 | 75,707 |

Corresponding numbers in geometrical proportion—

| | | | | | | | |
|---|---|---|---|---|---|---|---|
| | *Autumn.* | | *Spring.* | | *Summer.* | | *Winter.* |
| Marriages, | 36,306 | : | 31,355 | : : | 29,570 | : | 25,537 |
| | *Winter.* | | *Spring.* | | *Autumn.* | | *Summer.* |
| Births . | 131,257 | : | 129,677 | : : | 121,437 | : | 119,975 |
| | *Winter.* | | *Spring.* | | *Autumn.* | | *Summer.* |
| Deaths . | 97,765 | : | 89,141 | : : | 83,335 | : | 75,983 |

The seasons have most influence on the number of marriages; least on the number of births. If 100 be taken to represent the lowest average number registered in a quarter, the births rise to 108, the marriages to 142, the deaths to 129. According to the abstracts down to the present time, the births and deaths are most numerous in Winter, marriages in Autumn; whilst the smallest number of births and deaths occur in Summer, of marriages in Winter. It is a matter which has been frequently observed, that the marriages and births are most numerous where the mortality is highest.

We were very desirous of presenting to our readers an account of the construction and uses of *Life-Tables*, as they are called, into the nature and method of which the Registrar-General has entered very fully. Fearing, however, that such details would be rather foreign to the pages of a Journal, whose duty and character it is to be practical, we shall content ourselves with stating some of the more prominent applications of these tables. A Life-Table shows, out of a given number born alive, the numbers living at every year of age, for 100 or 105 years. The assumed number born alive, technically called the *base* or *radix* of the table, is arbitrary; and the age at which the table terminates varies in different tables. The yearly deaths are called the "*decrements of life.*" In the Life-Table given in this volume it is assumed that 100,000 form the *basis;* from the proportion of the two sexes registered, it will be found that 51,274 of them were boys, 48,726 girls. In order to facilitate the understanding of the principle, we will further assume that the 100,000 were all born on the same day—the 1st of Jan. 1841; and that the survivors, counted on the first day of 1842, 1843, and of every year for the next 100 years, will exist in the numbers against the respective ages of the annexed table. Now, on inspecting the table, it will be seen that out of the 100,000 children born on Jan. 1st, 1841, only 85,369 were alive on Jan. 1, 1842; so that 14,631 perished in the first year. Jan. 1, 1843, the survivors were two years old, and in number 80,102; so that 5,267 died in the second year. Jan. 1, 1846, the 5th year of age will be attained, and there will be 74,201 living. So that in the first five years 25,799 of the 100,000 children born, die. During the next five years, the mortality becomes less considerable; and from 10 to 15 the loss of life remains small—68,627 will live to the age of 15. At this age the loss of life among girls is greater than among boys, and it continues so for the next five years. The mortality appears to increase rather rapidly from 12 to 15; and then at a slow regular rate from 15 to 55 years: 66,059 attain the age of 20. It was observed that 51,274 boys were born alive to 48,726 girls; but the mortality in infancy is greater among boys than girls; so that 31,958 males attain the age of 25, and 31,623 females attain the age of 24. This is about the average age of marriage in England; and the number of the two sexes is then nearly equal. About four-fifths of the males who attain the age of manhood marry; the proportion of women who marry being the same. Of the 100,000 persons born, 50,301 attain the age of 45, viz. 25,311 men, and 24,990 women. The chance of living from 25 to 45 is rather in favour of English women. The various hardships, perils, and annoyances to which men are exposed counterbalance the dangers of child-bearing. At the age of 55 a more rapid rate of mortality will set in, and more than a thousand die every year; yet 37,996 will be alive at the age of 60, and 24,531 attain the age of 70—11,823 men, and 12,708 women—the mortality of women being less than that of men after 55:—by the various causes of wear and tear, and more than all, by the natural falling off of vitality, at the age of 80 the numbers will be reduced to 9,398. After the age of 80 the observations grow uncertain; but if we admit their accuracy, 1,140 will attain the age of 90; 16 that of 100; and of the 100,000, one man and one woman—like the lingering barks of an innumerable convoy—will reach their distant haven in 105 years, and die in 1945. The nature of this calculation may be readily understood. It may have been ascertained that of 100,000 children born in Jan. 1841, 80,102 were alive in Jan. 1843; but we could not, of course, if so disposed, know by *direct means* how many will live through the year, and see 1844; it was, however, ascertained at the census that there were 437,276 children living in 1841 of the age of 2 and under 3 years; the deaths of 15,027 children of the same age were registered. Hence as 15,027 died to 437,276 living, it is a mere matter of numbers to determine how many die and how many survive a year out of 80,102 children exactly 2 years old. According to the Table, 2,710 die out of 80,102, and 77,392 attain the third birth-day, and will be alive Jan. 1, 1844.

The mortality at certain intervals of age can always be determined from a comparison of the numbers living with the deaths: and from the ascertained mortality the annual survivors can be calculated. Thus in 1841 it was found that 6,633 men died at the age 20—25 out of 724,013 living; the mean age of those persons may be taken to be $22\frac{1}{2}$ years: we know the mortality, therefore, at that age, and can tell how many of a given number, say 32,792, aged 22, will live a year—how many of the 100,000 alive on Jan. 1, 1863, will be alive on Jan. 1, 1864.

The Life-Table, in its present form, as given in the volume now under consideration, possesses several remarkable properties. It shows the *probability or chance of living a year or any number of years at any age.* Thus 100,000 forming the *base* or *radix,* and 85,369 being the number living to the end of the first year, at birth the chance of living a year is .85,369, the chance of dying .14,631; for there are 100,000 chances, and 85,369 in favour of living. At the age of 40 the chance of living a year is $\frac{53,134}{53,825}$; for, according to the Table, the number who die in the next year is 691, and the number who survive is 53,134; so it is 53,134 to 691, that a person aged 40 will live a year. At 60 the chance of living a year is $\frac{36,874}{37,996}$; the denominator of the fraction (37,996) expressing the total number of chances, and the numerator (36,874) the chances in favour of living. The chance of dying is $\frac{1,122}{37,996}$, and the two fractions added together $\frac{36,874 + 1,122}{37,996} = \frac{37,996}{37,996} = 1$: unity being in the arithmetic of probabilities the symbol of certainty, the certainty that the person will die or live, is thus expressed.

By inspecting this Table we may readily see the probable duration of life; it is in fact the time in which the number born is reduced one-half; in the English Life-Table this will be found to be $45\frac{1}{2}$ years; that is, it is probable that a child will live to $45\frac{1}{2}$ years: for, by looking at the Table, it will be seen that the 100,000 are reduced to 50,301—nearly half their number—by the age 45; there is, therefore, nearly an equal number of chances (50,000) in favour of living to, and of dying before the age 45½. The *probable* life of a boy is 44, of a girl 47 years. Suppose we wish to ascertain how long it is probable that a woman aged 25 will live. On inspecting the table, we see that the survivors out of the 100,000, who have attained their 25th year, are 31,337; the half of this is 15,668, the number surviving at the age of 66; it is *probable*, therefore, that the woman will live to the age of 66, that is, that she will live for 41 years after her 25th year. Again, suppose we wish to ascertain the age of a man at the age of 60. By inspecting the table we see that the number of survivors at this age, is 18,808; now the half of this is 9,404, to which the 18,808 are seen in the table to be reduced at the age of 73; at 60, therefore, it is *probable* that a man will yet live 13 years.

Several uses may be made of the Life-Table. It may be readily converted into a population table, shewing the total numbers living, and the numbers living at every age. The uses of the Life-Table in determining the value of life annuities, leases, livings, pensions, &c. are well known. We are almost tempted to present to our readers one or two instances of the method of employing it in such cases, but we apprehend it may lead us from our more legitimate department.

By adding up the column of "LIVING," as given in the English Life-Table now under consideration, the sum of the numbers will be found to amount to 4,165,890; subtract half 100,000 from this, and 4,115,890, the number of the years which the 100,000 persons live, will be obtained. Divide the years of life, 4,115,890, by 100,000, and the quotient, 41.16, will be the mean age, or, as it is sometimes called the *expectation of life;* for males it is 40 years, females 42, and for both sexes 41 years. By repeating the process, the expectation of life at each year of age is obtained; at five years, it is 50 years; at ten 47; at twenty 40; at thirty 34; at forty 27; at fifty 21; at sixty 14, &c. &c. The average age at which persons aged 30 will die, is 64 years, and 74 is the average age at which sexagenarians will die.

It is observed, that one of the most remarkable points in the comparisons of the expectation of life at different times, in different nations, and various climates, is their singular uniformity. This uniformity does not imply that the external circumstances in which men live have no influence on the duration of life; it only tends to prove that life being regulated by constant laws, the circumstances, adverse or favourable to existence produced, by compensations of various kinds, the same results. The mortality of England varies from year to year; and the mortality of 1841 was rather lower than in previous years; so this table is only given as an approximation to a mean table. We shall now proceed to consider the contents of Mr. Farr's three papers on subjects connected with the Abstracts in the Registrar-General's Annual Report. The first of these papers treats of the construction of Life-Tables. The second presents a general view of the fatal diseases of the year 1841. The third, in continuation of previous papers, is devoted to the examination of the diseases of towns, and their causes. As we have already allotted a considerable portion of space to the subject of Life-Tables, we shall pass on to the second of Mr. Farr's papers, concerning the *Public Health*. The number of deaths registered in the year 1841 was 343,847, less by 15,714 than

359,561, the number registered in 1840. The mortality was .001,289 less in 1841 than in the previous year. In 1840 out of a million living 22,878 persons died; in 1841 out of the same number living 21,589 died. Of the decrease, 897 in the 1,289 was in the *zymotic* class of diseases. The idea attached to the term *zymotic*, Mr. Farr has already explained in his Appendix to the Fourth Report, where he states that he considers a designation like this, as more convenient than the periphrasis "epidemic, endemic, and contagious diseases." It is a property of zymotic diseases to prevail more at one time than at another; to become epidemic, or endemic, or contagious in certain circumstances. The epidemic character of the diseases of this class has been exemplified very remarkably by scarlatina, which raged with increasing severity until 1840, and continued the prevailing epidemic of 1841, though it had begun to decline: 14,161 persons, principally children, died of scarlatina in 1841; and 19,816 had died by this malignant malady in 1840. Measles had been epidemic and destroyed 10,935 lives in 1839; the deaths from measles in 1841 were 6,894. Small-pox was fatal to 16,268 persons in 1838, to 9,131 in 1839, to 10,434 in 1840, and to only 6,368 in 1841. It was less fatal in 1841 than either measles, scarlatina, or hooping-cough. This happy result is probably to be ascribed, in a great part, to the Vaccination Act, which came into operation in 1840; and may be expected, by extending the application of Jenner's great discovery, to effect a still further reduction in the sufferings, deformity, and mortality dependent on small-pox.

The deaths of 48,053 persons were ascribed to diseases of uncertain or variable seat. Many of the cases of "hæmorrhage," returned "rupture of a blood-vessel," &c. belong to phthisis, to which head hæmoptysis, or spitting of blood, was referred. Diseases of the respiratory organs were fatal to 92,183 persons in 1841; the mortality which they occasioned was nearly six in 1,000; it was 5,911 in a million, or 132 less than in 1840, when 6,043 in a millon died of pulmonary affections. Of the decrease of 132 to a million 54 was in pneumonia, and 75 in phthisis. The mortality by these two diseases remained, nevertheless, excessively high: thus—

| | 1840 | 1841 |
|---|---|---|
| Total deaths by Pneumonia .. .. .. .. | 18,582 | 17,997 |
| „ deaths to a million living .. .. .. .. | 1,209 | 1,154 |

| | 1840 | 1841 |
|---|---|---|
| Total deaths by *Phthisis* .. .. .. .. .. | 59,923 | 59,592 |
| „ deaths to a million living .. .. .. .. | 3,897 | 3,822 |

The progressive increase in the number of cases registered as heart disease, from 3,319 to 4,246, may be ascribed to the diffusion of new methods of diagnosis, as well as to improvements in the character of the registration.

The deaths by diseases of the digestive organs were 22,398. The mortality was 1,436 in a million, or 29 less than in the previous year.

*Deaths by Childbirth.*

In 1841, 3,007 mothers died in childbirth. On an average 8 died from childbirth every day of the year. The mortality was one death to 171 births registered.

We entirely concur in Mr. Farr's remarks on the serious advantages which must result from the establishment of schools for the education of nurses and midwives in the metropolis and large towns. By such means a highly useful profession would be thrown open to women; and the utility to the community of a recognised body of respectable women, educated as nurses, acquainted with the plain doctrines laid down in the popular medical works on health, and possessing as much knowledge of midwifery as the French *sage-femme*, would be incalculable. Some of these schools, he thinks, might be connected with the present hospitals and lying-in institutions; others might be founded for the delivery of easy popular lectures, and for providing the wives of the indigent with gratuitous attendance, or attendance slightly remunerated—to be supplied by the young nurses, superintended by those practically versed in their art, and by medical officers. In a year or two years, intelligent women would acquire, at such an institution, sufficient information and skill to be useful nurses. He thinks it questionable, however, whether they should be taught the properties of drugs. He thinks it would be quite enough if they were taught in what circumstances to give a few drops of laudanum after delivery, and when to administer castor oil, or tincture of rhubarb, themselves, or in what way to apply the remedies prescribed by physicians or surgeons. To do more, would be to establish a new class of half-educated practitioners, like the druggists, and would infallibly lead to mischief, without any chance or prospect of countervailing good. The suggestions thrown out by Mr. Farr, in this part of his paper, are truly valuable, and well deserving the attentive consideration of the community at large. After having consulted several medical men in extensive practice, he states, that the want of good educated, trustworthy nurses, is felt in the highest circles, as well as in the middle ranks of society. He conceives that an institution for the education of nurses would probably succeed better than many of the medical schools; but they would be nurses for the middle and higher classes; the small outlay of capital which an education of the kind would involve, must tend very much to preclude the admission of midwives for the poor labourer's or the artizan's wife. For the many collateral advantages that would result to society from the possession of well-educated nurses we must refer to the original paper.

The next most important subject discussed in Mr. Farr's paper, is the *Causes of the High Mortality in the Town Districts.*

The population, in 1841, of the districts in the counties of Essex, Norfolk (except Norwich), Suffolk, &c. &c. an area of about 9,352 square miles, was 1,700,484. The deaths registered in the 4 years (1838-41) were 132,116.

The population of Birmingham, Aston, Bristol, Clifton, and several other towns, standing on 666 square miles, was 1,883,693, and the deaths registered in the 4 years were 205,966. The population of the town was greater than that of the country districts by 183,209; as a correction for this excess strike off 20,000 from the deaths in the former districts, and the excess of the deaths in the town districts will be found to be 53,850.

For another comparison the Metropolis, inhabited in 1841 by 1,875,493 persons, was compared with the South Western Division, comprising in Wiltshire, Dorsetshire, Devonshire, Cornwall, Somersetshire, a population of 1,740,017. The deaths were 189,927 in the Metropolis, 130,298 in the South Western Division, and after the same kind of correction as above, the result is of the same character.

The essential character, by which Mr. Farr was guided in classifying districts under the head of "town" or "country," is the density of the population, which can be expressed numerically by the "population to a given area," or the "area to each person." After having obtained from an immense number of facts the undeniable result, that certain diseases are much more fatal, and that the mortality is much greater, in towns than in the open country, Mr. Farr proceeds to enquire into the causes of the diseases, which the registration has proved to be so much more fatal in the town than in the country districts. To distinguish those causes, and to determine precisely the share which they have, single or combined, in the production of disease, it becomes necessary to institute an elaborate series of observations and experiments, in which the resources of chemistry, natural philosophy, medicine, and the mathematical sciences, must be called into requisition. Our present information does something more than prove the necessity of further inquiry; it enables us to trace the disease of towns to groups of causes, and to analyze them partially. And first with respect to the necessaries of life, a deficiency of which may stand as one of the causes of diseases in towns; these being the produce of labour, and of variable value, a portion of every population, savage or civilized, is unable to procure them, and is subject to privation in different degrees. These necessaries are, 1, Drink; 2, Food (animal and vegetable); 3, Physic; 4, Clothing; 5, Firing; 6, Lodging; 7, Cleansing. The relative value of these articles is represented by their price, which varies at different times and places; this price, however, it is evident, does not express the relative facility of procuring them; such facility being expressed by the ratio of the earnings of a family to the cost of its subsistence. Now when in any case this ratio is one of equality, there is what may be called a competency. This may be expressed by the value of the above-mentioned ratio. Let us suppose that a sufficient supply of necessaries can be procured by a family for £100. per annum; what would be the effect of reducing the earnings to £50.? Experience proves to us, that the duration of life is not diminished in that ratio, for this reason, that by substituting a cheaper food the body may be sufficiently nourished. A low standard of subsistence, however, might be fixed on, any fall below which would be accompanied by a certain reduction of the mean life of the people. The ratio may be conceived under this simple form—

$$\frac{\text{Income}}{\text{Drink} + \text{food} + \text{physic} + \text{clothing} + \text{firing} + \text{lodging} + \text{cleansing},} = r;$$

and putting N for income (in money), C for the aggregate cost (in money) of the seven necessaries, and L for the mean physiological duration of life, the equation is $\frac{N}{C}L = L'$; L' being the mean life in the particular circumstances. Suppose the cost of a full supply of subsistence for a labourer in the country to be £50. a year, and that the income of his family is £50. and that he is in such favourable circumstances altogether that he attains the natural mean term of life, the equation will be $\frac{50}{50}L=L$. But let the income be reduced to £40, and if the ratio be simple the equation will be $\frac{40}{50}L=\frac{4}{5}L$. So that, if the mean natural life $=56$ years, it would be reduced to $\frac{4}{5}56$, or 45 years, which is the present mean life in the best country districts. So that, by having several series of observations on the incomes of the labouring population in town and country, in different circumstances, together with the cost of the necessaries of life, we might determine the effect on the mean life of the people. It is hoped that such observations will be made. There are circumstances, however, which must prevent us from admitting that the command over the necessaries of life is in favour of the country population.

Another class of causes which may be supposed to affect the mean duration of life consists of atmospheric impurities, organic matter undergoing decomposition, and the contagious principles of zymotic diseases. The most minute investigation has established the fact, that the excessive mortality in towns is scarcely in any degree to be ascribed to the accumulation of carbonic acid gas, or any other gases, so complete is the provision made for their dispersion. The next consideration is, whether it is to matters suspended in the atmosphere of cities that the excessive mortality must be referred. Smoke is heated gas, carrying with it unburnt particles in suspension; the carbonic acid is scattered immediately by its diffusive velocity, and the particles of solid matter, carried up by the heated air into the sky, disperse, become invisible, and fall around insensibly, in a clear atmosphere, or at a distance when there is any wind. That the smoke is irritating to the air-passages, injurious to the health, and one of the causes of death, to which the inhabitants of towns are more exposed than the inhabitants of the country, is exceedingly probable; but, if the effect were very considerable, it would be most evident in the dense fogs, when the atmosphere is loaded with smoke, and breathed for several consecutive hours by the population—men, women, and children. Now no connexion has ever been observed between the increase of the mortality and the London fogs. The diseases, again, caused by smoke, must be of a mechanical nature, and affect the lungs and air-passages; it may increase the pulmonary diseases, but will assuredly not produce scarlatina, measles, typhus, and other diseases which prevail in towns. There is still another class of agents. Suppose in a school-room there are 100 children: a child is introduced for a few hours, in a state of scarlatina. The children have not had the disease before; 10 of them are affected. If 10 children with scarlatina were introduced, and the room were ill-ventilated, 30 or 40 of the children might be affected. The same may be said of other contagious diseases, as small-pox, measles, hooping-cough, dysentery, &c. &c. The numbers attacked by an infectious disease depend upon—1st. the susceptibility of the persons exposed; 2ndly, on the strength of the zymotic matter, which varies in the stages and forms of the several diseases; and 3rdly, on the density of the air in the room, and on the ventilation in it. If 100 healthy were placed in a room in immediate contact with 10

sick persons, if the room were small, the doors and windows closed, the greatest number possible would be infected; and if they went through the disease in the same circumstances, the mortality would also be the greatest possible. Thus, in consequence of the various public buildings in which crowds of persons assemble not being provided with the proper mechanical means of ventilation, and the air not being withdrawn, the very walls reek with the breathed atmosphere; and if any epidemic, such as influenza, be rife, several persons affected with the complaint are present, and great numbers are infected; the headache and oppression which come on are the first and often not the last symptoms. This is literally "taking poison;" though generally called "taking cold." It is a vulgar error to suppose that rooms are healthy when they are not hot; but the heat which is generated may increase the effect of the zymotic matter. Certain substances, then, taken from the bodies of the sick, produce, when introduced into other bodies, a series of specific phenomena, developed according to a determined type. *Varioline,* for instance, produces small-pox. These substances act as ferments.

The 267 feet of air passing through the lungs daily, if charged with these particles, will bring them into contact with the blood. With respect to the physical properties and chemical nature of these morbific particles, Professor Graham's hypothesis is, that they are highly organised particles of fixed matter, which may find its way into the atmosphere, notwithstanding, like the pollen of flowers, and remain for a time suspended in it; a condition which is consistent with the admitted difficulty of reaching and destroying those bodies by gaseous chlorine, and with the washing of walls and floors as an ordinary disinfecting practice.

It is certain that animal matter is exhaled from the lungs and cutaneous surface. The particles are small and rare, but inelastic, devoid of the diffusive force of gases and vapours. These particles, so held in suspension and inelastic, stagnate in air, and can only fall to the ground, or be carried away with the fluid in which they float. Smoke and organic matters are removed from a room in this way—by replacing all its gaseous contents. Every population throws off insensibly an atmosphere of organic matter, excessively rare in country and town, but less rare in dense than in open districts; and this atmosphere hangs over cities like a light cloud, slowly spreading—driven about—falling—dispersed by the winds—washed down by showers. This is matter which *has lived,* is now dead, has left the body, and is undergoing by oxidation decomposition into simpler than organic elements. The exhalations from sewers, churchyards, vaults, slaughter-houses, cess-pools, commingle in this atmosphere as polluted waters enter the Thames; and, notwithstanding the great provisions of Nature for the speedy oxidation of organic matter in water and air, accumulate, and the density of the poison (for in the transition of decay it is a poison) is sufficient to impress its destructive action on the living—to receive and impart the processes of zymotic principles—to connect by a subtle, sickly, deadly medium, the people agglomerated in narrow streets and courts, down which the wind does not blow, and on which the sun seldom shines. A small quantity only of organic matter can escape with the carbon and aqueous vapour from the skin and lungs (37½ ounces daily, according to Dalton). Liebig, by operating on large masses of air, has obtained ammonia, which is a product of the putrefaction of animal matter. So that the existence in the atmosphere of organic matter is incontestable; and as it must be most dense in the densest districts, where it is produced in greatest quantities, and the facilities for its decomposition and removal are least, its effects—disease and death—will be most evident in towns. To this cause it is that Mr. Farr ascribes the mortality of towns, the people living in an atmosphere charged with decomposing matter, of vegetable and animal origin. He next proceeds to prove, not only that the mortality is greater in the town than in the country districts, but that the mortality of town districts has a certain relation to their density. The relation exists strictly between the density of the organic particles suspended in the atmosphere and the mortality; the density, however, of the matter in the air cannot be determined directly; and with the same number of persons on a square mile, the number of particles in the atmosphere will vary in different districts, according as the means of removing the refuse matter, by sewerage and other means, are more or less efficient. It is evident that, from the occupations of men in towns being mostly carried on in-doors, and in crowded workshops, while the agricultural labourer spends the greater part of the day in the open air, that the artisan in-doors is not placed in the same favourable circumstances as the labourer; the sun and wind carry off the breath as soon as it is formed, while in the workshops of towns the men are shut in from the sun, and no streams of air carry off the steaming breath and perspiration; hence the mortality of working men in the Metropolis is much greater than the mortality of women at the corresponding ages—no such great discrepancy existing in the country districts. Thus, in estimating the effects of density of population in districts, Mr. Farr takes examples from the observations on females, in order to simplify the enquiry. The Metropolis presents the most favourable opportunity for investigating the influence of density; it is an aggregate of masses of people in districts larger, more homogeneous, and more on an equality in respect to subsistence, than could be found elsewhere; the difference between district and district is very great; but yet districts may be selected resembling each other on an average in all other points, save density.

For the purpose of comparison, he selects three of the statistical districts of the metropolis in which the mortality is highest—Whitechapel, Shoreditch, and Bethnal Green. These resemble each other in a great many particulars; but the density is different, and the mortality is greatest in the densest districts.

| DISTRICTS. | Annual Mortality of Females (*m.*) | Population to a Square Mile (*d.*) |
|---|---|---|
| Whitechapel .. .. | ·02978 | 127,313 |
| Shoreditch .. .. | ·02790 | 86,123 |
| Bethnal Green .. | ·02617 | 62,390 |

The mortality here taken was for the four years 1838—41.

By mere inspection of this table it is evident that where the density is greatest, the mortality is also greatest—now the density of population in Whitechapel is as great and half as great again as it is in Shoreditch; that is, the density of population in Whitechapel, is to the density in Shoreditch nearly as three to two. The mortality, however, of these two districts is not in this ratio—representing the density of the densest district (Whitechapel) by $d^1$, that of the less dense (Shoreditch) by $d$; the mortality of the densest district (Whitechapel) by $m^1$, and that of Shoreditch by $m$, the proportion does not hold:—

$$m^1 : m :: d^1 : d.$$

On reducing the terms to the form of an equation, it will be seen that the mortality increased as the sixth roots of the densities: for $\frac{m^1}{m} = \sqrt{\frac{d^1}{d}}$ and consequently $m^1 : m :: \sqrt[6]{d^1} : \sqrt[6]{d}$.

To facilitate the understanding of this, to those not versed in the language of algebra—let us suppose the density of Whitechapel ($d^1$) to be expressed by the number 15,625, that of Shoreditch, by the number 729; the mortality of one district will not be to that of the other as 15,625* to 729; but as the sixth root of 15,625 = 5, to the sixth root of 729 = 3; that is as 5 : 3.

From the above equation we have the formula $m^1 = \sqrt[6]{\frac{d^1}{d}}\, m$, that is, we have the mortality of Whitechapel expressed in terms of the mortality of Shoreditch, and the densities of Shoreditch and Whitechapel. By substituting the numbers expressing the mortality of Whitechapel, and the densities of Whitechapel and Bethnal Green in the formula, we obtain the equation $\sqrt[6]{\frac{62,390}{127,313}} + \cdot 02978 = \cdot 0264$ for the mortality of Bethnal Green, which almost entirely coincides with that mortality, as ascertained by direct observation, which is ·0262.

To ascertain whether the mortality bears the same ratio, the density in the wealthiest districts of the metropolis, Mr. Farr selects the following districts; *scil.* St. James's, City of London, and St. George, Hanover Square.

| DISTRICTS. | Annual Mortality of Females (*m.*) | Density of Population. |
|---|---|---|
| St. James's .. .. | ·02145 | 145,059 |
| City of London .. | ·01968 | 94,488 |
| St. George, Hanover Square .. .. | ·01707 | 39,018 |

* These numbers, 15,625 and 729, have been selected as being the even sixth powers of the simple numbers 5 and 3.—*Rev.*

Now given the mortality of St. James's district = ·02145; the density of population 145,059; the density of St. George's, 39,018, to determine the mortality of St. George's.

Here we again employ the former formula, *scil.* $m = \sqrt[6]{\frac{d^1}{d}}\, m^1$—and substituting the figures for the corresponding algebraic characters, we have $\sqrt[6]{\frac{39{,}018}{145{,}059}} + {\cdot}02145 = {\cdot}0172$, the computed mortality of St. George, which nearly coincides with that ascertained by direct observation, *scil.* ·01707.

By the same formula the density of a district may be obtained.

Thus, then, it is proved beyond a doubt, that if the population be the same in other respects, an increase of density implies an increase of mortality; and that the ratio of increase is as certain roots of the density. When a more extended enquiry has confirmed this principle, *scil.* that the ratio of mortality is as the sixth roots of the densities, it will be time to ask why this should be the particular ratio. But the chemists must first discover the means of determining the density of the *zymotic atmosphere*, in different districts; for the density of the population is no strict measure of the density of this zymotic atmosphere; nor, admitting that the matter is a poison, does the relative density of the population express the relative doses inhaled in a given time; if it did, it is improbable and contrary to all analogy that the mortality should increase in the simple ratio of the dose. The exact effect of increasing doses of poison, has not been accurately determined; but it is well known that small doses of all poisons are taken with impunity, and that the dose of arsenic, opium or prussic acid may be increased up to a given point, at which the disease produced is severe or fatal. Four drops of prussic acid diluted, may be taken with safety, when four drops more would be fatal to a certain number of persons. How small the dose of matter may be which will produce a zymotic disease, it is impossible to say; but, if a minute diluted charge of *vaccinine* (vaccine lymph) produce cow-pox, say one time in 1,000, it would be an interesting problem to determine, by doubling the quantity, in what ratio the proportions infected increased.

Mr. Farr having shewn, by a multitude of facts, contained in this paper, as also by accompanying tables, the necessity of changing the atmosphere of towns, as rapidly as possible, and of diluting it to the greatest possible extent, next offers some valuable suggestions with respect to the diminution of the quantity of animal and vegetable matter thrown into the atmosphere of towns. This matter is derived from several sources;—the halitus of men and animals, the soil of water-closets, burial-grounds, slaughter-houses, sewers, and streets. The *halitus* cannot be diminished in quantity, but the isolation of families in separate houses tends to prevent its accumulation. For valuable and excellent remarks on the danger of assembling large numbers of sick into the same buildings, as in the instance of large hospitals, we must refer to Mr. Farr's paper. It is well known that when hospitals are crowded, the increase of mortality soon becomes striking—the mortality in the large metropolitan hospitals is twice as great as in the small country hospitals. The patient is fortunate who escapes phlebitis or purulent deposits, after any serious operation in an hospital. Mr. Farr observes, further, that the space allotted for the sleeping-rooms of many public institutions in towns is too small. It should be in no case less than eight feet cube (= 512 cubic feet) to each person, with proper apertures for the removal of the breath. The influence of a too limited space, if carefully investigated, would no doubt show a certain inverse relation between the mortality and the space, a death marking every degree of concentration of the expired atmosphere. From the extent to which we find we have carried our analysis of this Report, we must now conclude, earnestly recommending its attentive perusal to the medical profession. It abounds in suggestions and advice of the most valuable and practical kind—and reflects the highest credit on the judgment and great scientific attainments of the gentleman engaged in drawing it up.

